# Supplementary material for: Determination by ICP-MS of Essential and Toxic Trace Elements in Gums and Carrageenans Used as Food Additives Commercially Available in the Portuguese Market
Source: Foods. 2023 Mar 26;12(7):1408. doi: 10.3390/foods12071408 (PMC10093682; doi:10.3390/foods12071408)
Supplement: Supplementary file 1 [file foods-12-01408-s001.zip › foods-2252325-supplementary.pdf]

Determination by ICP-MS of Essential and Toxic Trace Elements in Gums and Carrageenans Used as Food Additives Commercially Available in the Portuguese Market.

Rui Azevedo, Ana Rafaela Oliveira, Agostinho Almeida and Lígia Rebelo Gomes

Table S1. Analytical quality control - Results of certified reference materials analysis (μg/g).

BCR-679 Cabbage Powder																
	Li	Al	Cr	Mn	Fe	Co	Cu	Zn	As	Se	Rb	Sr	Mo	Cd	Hg	Pb
**Mean**	0.068	< LQ	0.60	13.3	66	0.090	2.651	74.1	< LD	0.125	2.276	11.96	16.50	1.572	< LD	0.378
**Standard Deviation**	0.003	-	0.10	0.5	2	0.013	0.013	1.0	-	0.019	0.032	0.20	0.76	0.015	-	0.015
**Analytical Value**	-	-	0.60	13.3	55	-	2.89	79.7	-	-	-	11.8	14.8	1.66	0.006*	-
**Uncertainty**	-	-	0.10	0.5	2.5	-	0.12	2.7	-	-	-	0.2	0.5	0.07	0.001	-
**Recovery**	-	-	93%	104%	120%	-	92%	93%	-	-	-	101%	112%	95%	301%	-

*Inferior to the limit of detection (LD).

BCR-482 Lichen Powder																
	Li	Al	Cr	Mn	Fe	Co	Cu	Zn	As	Se	Rb	Sr	Mo	Cd	Hg	Pb
**Mean**	0.665	1001	3.67	27.2	668	0.297	5.77	75.4	0.763	0.521	7.65	8.699	0.372	0.478	0.455	38.1
**Standard Deviation**	0.015	38	0.11	1.0	25	0.017	0.17	2.3	0.053	0.029	0.23	0.082	0.017	0.055	0.018	2.3
**Analytical Value**	-	1103	4.12	33.0	804	0.32	7.03	100.6	0.85	0.60	-	-	0.85	0.56	0.48	40.9
**Uncertainty**	-	24	0.15	0.5	160	0.03	0.19	2.2	0.07	0.20	-	-	0.01	0.02	0.02	1.4
**Recovery**	-	91%	89%	82%	83%	93%	82%	75%	90%	87%	-	-	44%	85%	95%	93%

Table S2. Limits of detection (LD) and limits of quantification (LQ; μg/g).

	**Li**	**Al**	**Cr**	**Mn**	**Fe**	**Co**	**Cu**	**Zn**	**As**	**Se**	**Rb**	**Sr**	**Mo**	**Cd**	**Hg**	**Pb**
**LD**	0.018	5.1	0.025	0.029	0.23	0.002	0.033	0.034	0.047	0.022	0.008	0.081	0.006	0.003	0.009	0.007
**LQ**	0.053	15.5	0.074	0.086	0.69	0.005	0.099	0.10	0.14	0.067	0.023	0.24	0.018	0.009	0.028	0.020

The limits of detection (LD) and quantification (LQ) of every element were obtained by the following formula, respectively: $SD_{blanks} \times 3.3$ and $SD_{blanks} \times 10$
